# Relationship Between Orthostatic Hypotension and Cognitive Functions in Multiple System Atrophy: A Longitudinal Study

**DOI:** 10.3389/fneur.2021.711358

**Published:** 2021-09-03

**Authors:** Sofia Cuoco, Immacolata Carotenuto, Arianna Cappiello, Sara Scannapieco, Maria Claudia Russillo, Valentina Andreozzi, Lorenzo Forino, Marianna Amboni, Marina Picillo, Roberto Erro, Paolo Barone, Maria Teresa Pellecchia

**Affiliations:** Center for Neurodegenerative diseases (CEMAND), Department of Medicine, Surgery and Dentistry, Neuroscience section, University of Salerno, Salerno, Italy

**Keywords:** α-synucleinopathy, cognitive dysfunction, global cognitive status, multiple system atrophy, orthostatic hypotension

## Abstract

**Introduction:** The aim of this study is to investigate the impact of orthostatic hypotension (OH) on cognitive functions in patients with multiple system atrophy (MSA) followed over time.

**Methods:** Thirty-two MSA patients were enrolled and underwent a comprehensive neuropsychological battery; at baseline (T_0_) 15 out of 32 patients presented OH, assessed by means of orthostatic standing test. All patients underwent a follow-up (T_1_) evaluation 12 months after baseline. Thirteen out of 32 patients also underwent a second follow-up (T_2_) evaluation at 24 months. Changes over time on different neuropsychological tasks were compared between patients with and without OH by means of Mann-Whitney's *U*-test. Moreover, clinical categories of normal cognition, mild cognitive impairment, and dementia were determined, and changes at T_1_ and T_2_ in global cognitive status were compared between patients with and without OH.

**Results:** At T_0_, patients with OH had better performance on words/non-words repetition task (*p* = 0.02) compared to patients without OH. Compared to patients without OH, patients with OH performed worse on semantic association task (*p* < 0.01) at T_1_ and on Stroop test-error effect (*p* = 0.04) at T_2_. The percentage of patients with worsened cognitive status at T_1_ was higher among patients with OH than among patients without OH (93 vs. 59%, *p* = 0.03). OH (β = −4.67, *p* = 0.01), education (β = 0.45, *p* = 0.02), age (β = 0.19, *p* = 0.03), and Montreal Cognitive Assessment battery (MOCA) score at T_0_ (β = −0.26, *p* = 0.04) were significant predictors of global cognitive status worsening at T_1_.

**Discussion:** We found that global cognitive status worsened at 1-year follow-up in 93% of patients with OH, and OH, along with age, education, and MOCA score, predicted cognitive worsening over time. To clarify the relationship between OH and cognitive dysfunction in MSA, we suggest the use of clinical categories of normal cognition, mild cognitive impairment, and dementia in further longitudinal studies on MSA patients with and without OH.

## Introduction

Multiple system atrophy (MSA) is a sporadic, progressive α-synucleinopathy, clinically characterized by different combinations of rapidly progressive parkinsonism, cerebellar ataxia, autonomic failure, and corticospinal impairment. Specifically, the parkinsonian variant (MSA-P) is characterized by prominent akinetic-rigid parkinsonism and the cerebellar variant (MSA-C) by progressive ataxia (1, 2). In early disease stages, the diagnosis of MSA is mainly based on clinical features including significant autonomic dysfunction (3). One of the most important autonomic features of MSA is orthostatic hypotension (OH), defined as a blood pressure (BP) drop of at least 20/10 mm Hg (systolic/diastolic) from supine to standing position (4). OH results from cardiovascular dysfunction caused by a complex interplay between central and peripheral autonomic dysregulation, cardiac noradrenergic sympathetic denervation, peripheral norepinephrine deficiency, and arterial baroreflex failure, finally leading to impaired arterial vasoconstriction and reduced compensatory cardiac output in response to hypotension (4). The prevalence of symptomatic OH is 55–80% in patients with MSA (5, 6). The clinical manifestations of OH are generally insidious and significantly associated with syncope, presyncope, accidental falls (7, 8), and increased mortality among elderly patients (9). OH is usually associated with cognitive symptoms; indeed, confusion is often described by affected patients (7, 8), but there is poor evidence about the relationship between OH and cognitive deficits in MSA patients (10).

The aim of this study is to investigate the impact of OH on cognitive functions in a sample of MSA patients evaluated longitudinally by means of a comprehensive neuropsychological assessment.

## Methods

### Patients

Thirty-two patients with a diagnosis of probable MSA, according to current criteria (2), were enrolled at the Center for Neurodegenerative Diseases of the University of Salerno between November 2015 and April 2019. Brain magnetic resonance imaging (MRI) had been performed in all patients showing various combinations of putamen, middle cerebellar peduncle, pons, and cerebellum atrophy consistent with clinical diagnosis.

At baseline (T_0_) 15 of the 32 patients presented OH, defined by a reduction of systolic BP of at least 20 mm Hg or diastolic BP of 10 mm Hg within 3 min of standing (11). Symptomatic OH was present in 93% of patients presenting OH. Seventeen patients did not present OH at baseline and follow-up evaluations.

All patients underwent a follow-up (T_1_) evaluation 12 months after baseline. Thirteen out of 32 patients also underwent a second follow-up (T_2_) evaluation at 24 months. At T_2_ evaluation 4 patients were lost to follow-up, 8 were deceased, and 7 did not come back to the hospital due to Covid-19 restrictions. No patients received pharmacological treatment for OH during the study period.

The local Ethics Committee approved the study, and all patients signed informed consent.

### Clinical and Neuropsychological Assessments

Demographic and clinical features are listed in Table 1.

**Table 1:** Clinical, demographic, and neuropsychological features in MSA patient with and without OH at baseline.

	**MSA with OH at T_**0**_** **median (IQR)** **(*N* = 15)**	**MSA without OH at T_**0**_** **median (IQR)** **(*N* = 17)**	***p***
**Clinical and demographic variables**			
Age	58 (9)	62 (10)	0.96
Education	11 (11)	8 (4)	0.09
Disease duration	3 (3)	5 (2)	0.48
UMSARS-II	20 (7)	19 (16)	0.96
LEDD	375 (164)	304 (199)	0.60
	**MSA with OH at** **T**_**0**_% **(*****N*****= 15)**	**MSA without OH at** **T**_**0**_% **(*****N*****= 17)**	
Gender (M %)	47%	47%	0.63
Supine hypertension (%)	18%	0%	0.22
Urinary incontinence (%)	80%	88%	0.65
**Neuropsychological variables**			
**General cognition**			
- MOCA	21 (8)	21 (7)	0.07
**Memory domain**			
−15-RAWLT-recall^a^	7 (2)	7 (7)	0.39
- Prose memory^a^	12 (4)	10 (4)	0.66
- Recall of Rey Osterrieth figure^a^	10 (10)	15 (14)	0.84
**Attention domain**			
- TMT-A^a^	81 (48)	64 (88)	0.32
- Stroop test-error interference^a^	2 (3)	12 (18)	**0.04**
**Executive domain**			
- CDT^a^	9 (2)	10 (3)	0.19
- Verbal fluency test^a^	21 (17)	18 (23)	0.47
- Copy of the Rey Osterrieth figure^a^	28 (10)	33 (16)	0.52
**Visuospatial domain**			
- Constructional apraxia test^a^	11 (5)	11 (5)	0.48
- BJLO^a^	18 (8)	19 (11)	0.55
**Language domain**			
- Picture naming total^a^	12 (4)	13 (2)	0.98
- Words/no-words repetition-total^a^	8 (2)^b^	7 (2)^b^	**0.02**
- Semantic association^a^	4 (1)	4 (1)	0.12
**Mood domain**			
**- BDI-II**	13 (11)	15 (19)	0.95
**- AES**	33 (12)	36 (15)	0.20
**Functional autonomy**			
- ADL	5 (2)	6 (4)	0.84
- IADL^a^	4 (2)	6 (4)	0.86

*Statistically significant differences are indicated in bold*.

a *Tests used to identify NC, MCI, and dementia (12, 13)*.

b *MSA with OH vs. MSA without OH (corrected p ≤ 0.03)*.

*15-RAWLT, Rey's auditory 15-word learning test; ADL, Basic Activities of Daily Life; AES, Apathy Evaluation Scale; BDI-II, Beck Depression Inventory; BJLO, Benton's Judgment of Line Orientation; CDT, Clock Drawing test; IADL, Instrumental Activities of Daily Life; IQR, interquartile range; LEDD, total L-dopa equivalent daily dose; M, male; MCI, mild cognitive impairment; MOCA, Montreal Cognitive Assessment battery; MSA, multiple system atrophy; OH, orthostatic hypotension; N, number; NC, normal cognition; p, p-value; TMT-A, part A of Trail Making Test; T_0_, baseline; UMSARS, Unified Multiple System Atrophy Rating Scale*.

Cognitive abilities were assessed at baseline, T_1_, and T_2_ with the Montreal Cognitive Assessment (MOCA) (14), recall scores of the Rey's auditory verbal learning test (15-RAWLT-recall), recall and copy of Rey Osterrieth figure (ROCF), prose memory test, Trail Making Test-part A (TMT-A), Stroop Color-Word Test-error effect (SCWT), Clock Drawing test (CDT), verbal fluency test, constructional apraxia test, and Benton's Judgment of Line Orientation test (BJLO) (15), and sub-tests of Screening for Aphasia in NeuroDegeneration (SAND), such as naming, semantic association, and words and non-words repetition tasks (16). Depressive and apathetic symptoms were assessed by Beck Depression Inventory-II (BDI-II) and Apathy Evaluation Scale (AES) (17, 18).

Functional autonomy was evaluated with the Instrumental Activities of Daily Life (IADL) and with the Basic Activities of Daily Life (ADL). Where possible, tests were administered with random sequence.

### Statistical Analysis

After normality distribution check, using the Kolmogorov-Smirnov test, the differences in demographic and cognitive variables between MSA patients with and without OH at T_0_ were computed by χ^2^ or the Mann-Whitney's *U*-test.

Changes in neuropsychological scores between T_1_ and T_0_ were calculated (Delta = T_1_ – T_0_) and compared using Mann-Whitney's *U*-test in patients with and without OH. Changes in neuropsychological scores between T_2_ and T_0_ were calculated (Delta = T_2_ – T_0_) and compared using Mann-Whitney's *U*-test in patients with and without OH. Significant results obtained from the exploratory analysis were then corrected for multiple comparisons, where necessary.

Patients were also divided according to cognitive status in MSA with normal cognition (MSA-NC), MSA with mild cognitive impairment (MCI)-single domain, MSA with MCI-multiple domain, and MSA with dementia (MSA-D). Due to the lack of MSA-specific criteria for the diagnosis of MCI, it was diagnosed according to the Movement Disorders Society (MDS) criteria for MCI in Parkinson's disease (12). Dementia was diagnosed according to the Statistical Diagnostic Manual of Psychiatry-5th Edition (DSM-5). Subsequently, we divided the sample into two groups, according to worsening/not worsening of cognitive status at T_1_ and T_2_ as compared to T_0_, and we compared the percentage of patients with and without OH in the two groups, by χ^2^ and Fisher test, where necessary. Specifically, cognitive status was defined as worsening if at least one more cognitive domain resulted altered at T_1_ or T_2_ as compared with T_0_ evaluation.

A logistic regression analysis with bootstrap method was performed with worsening or not-worsening as the dependent variable and *p* < 0.05 deemed significant to evaluate a possible effect of OH, age, education, gender, disease severity, disease duration, and MOCA score at baseline on cognitive status change at T_1_. Due to the small sample size at T_2_, no logistic regression was performed.

Finally, we analyzed the changes in neuropsychological scores between T_1_ and T_0_ (Delta = T_1_ – T_0_) and between T_2_ and T_0_ (Delta = T_2_ – T_0_) among MSA-P and MSA-C patients separately, comparing patients with and without OH by means of Mann-Whitney's *U*-test. Significant results obtained from the exploratory analysis were then corrected for multiple comparisons, where necessary.

Statistical analyses were performed by SPSS-20 (SPSS Inc., Chicago, IL).

## Results

At T_0_, patients with and without OH did not differ by age, education, disease duration, disease severity, total L-dopa equivalent daily dose (LEDD), or gender (*p* > 0.05) (Table 1). At T_0_ patients with OH had better performance on words/non-words repetition task than patients without OH (*p* = 0.02) (Table 1).

At T_1_ patients with OH had worsened more than patients without OH on semantic association task (*p* < 0.01) (Table 2). At T_2_ patients with OH had worsened more than patients without OH on Stroop test-error effect (*p* = 0.04) (Table 3).

**Table 2:** Changes in neuropsychological tests in MSA patients with and without OH at T_1_.

	**MSA with OH** **median (IQR)** **(*N* = 15)**	**MSA without OH** **median (IQR)** **(*N* = 17)**	***p***
**Delta T**_**1**_ **– T**_**0**_			
**General cognition**			
- MOCA	−1 (4)	−1 (4)	0.86
**Memory domain**			
−15-RAWLT-recall^a^	−1 (4)	1 (6)	0.94
- Prose memory^a^	1 (3)	0 (5)	0.69
- Recall of Rey Osterrieth figure^a^	−1 (3)	−1 (9)	0.17
**Attention domain**			
- TMT-A^a^	0 (20)	8 (34)	0.60
- Stroop test-error interference^a^	−1 (3)	−4 (18)	0.23
**Executive domain**			
**-** CDT^a^	−1 (4)	−1 (4)	0.49
- Verbal fluency test^a^	−1 (5)	−1 (8)	0.63
- Copy of the Rey Osterrieth figure^a^	−5 (10)	−3 (12)	0.46
**Visuospatial domain**			
- Constructional apraxia test^a^	−1 (3)	−0 (3)	0.98
- BJLO^a^	0 (8)	−3 (4)	0.12
**Language domain**			
- Picture Naming total^a^	0 (2)	0 (3)	0.83
- Words/no-words repetition-total^a^	0 (3)	0 (1)	0.26
^−^ Semantic association^a^	−1 (1)	0 (0)	**<0.01**
**Mood domain**			
**- BDI-II**	1 (11)	3 (14)	0.14
**- AES**	3 (15)	3 (14)	0.48
**Functional autonomy**			
- ADL	−2 (2)	−1 (0)	0.15
- IADL^a^	−2 (3)	−1 (3)	0.88

*Statistically significant differences are indicated in bold*.

a *Tests used to identify NC, MCI, and dementia (16, 19)*.

*15-RAWLT, Rey's auditory 15-word learning test; ADL, Basic Activities of Daily Life; AES, Apathy Evaluation Scale; BDI-II, Beck Depression Inventory; BJLO, Benton's Judgment of Line Orientation; CDT, Clock Drawing test; IADL, Instrumental Activities of Daily Life; IQR, interquartile range; MCI, mild cognitive impairment; MOCA, Montreal Cognitive Assessment battery; MSA, multiple system atrophy; OH, orthostatic hypotension; N, number; NC, normal cognition; p, p-value; TMT-A, part A of Trail Making Test; T_0_, baseline; T_1_, follow up 1*.

**Table 3:** Changes in neuropsychological tests between MSA patients with and without OH at T_2_.

	**MSA with OH** **median (IQR)** **(*N* = 7)**	**MSA without OH** **median (IQR)** **(*N* = 6)**	***p***
**Delta T**_**2**_ **– T**_**0**_
**General cognition**			
- MOCA	−1 (5)	−4 (6)	0.66
**Memory domain**			
−15-RAWLT-recall^a^	1 (3)	−3 (7)	0.16
- Prose memory^a^	−1 (8)	0 (6)	0.86
- Recall of Rey Osterrieth figure^a^	−1 (3)	−4 (7)	0.17
**Attention domain**			
- TMT-A^a^	24 (26)	10 (39)	0.36
- Stroop test-error interference^a^	−1 (4)	−28 (29)	**0.04**
**Executive domain**
**-** CDT^a^	−2 (9)	−1 (4)	0.90
- Verbal fluency test^a^	−5 (14)	−3 (8)	0.93
- Copy of the Rey Osterrieth figure^a^	−7 (10)	−8 (19)	0.46
**Visuospatial domain**			
- Constructional apraxia test^a^	−2 (6)	−3 (3)	0.52
- BJLO^a^	−4 (7)	−4 (2)	0.92
**Language domain**			
- Picture Naming total^a^	0 (3)	−1 (3)	0.81
- Words/no-words repetition-total^a^	0 (0)	1 (0)	0.11
^−^ Semantic association^a^	−1 (2)	0 (0)	0.32
**Mood domain**			
**- BDI-II**	−4 (15)	−5 (6)	0.46
**- AES**	4 (36)	−5 (45)	0.48
**Functional autonomy**			
- ADL	−2 (1)	−2 (2)	0.62
- IADL^a^	−2 (3)	−1 (3)	0.64

*Statistically significant differences are indicated in bold*.

a *Tests used to identify NC, MCI, and dementia (16, 19)*.

*15-RAWLT, Rey's auditory 15-word learning test; ADL, Basic Activities of Daily Life; AES, Apathy Evaluation Scale; BDI-II, Beck Depression Inventory; BJLO, Benton's Judgment of Line Orientation; CDT, Clock Drawing test; IADL, Instrumental Activities of Daily Life; IQR, interquartile range; MCI, mild cognitive impairment; MOCA, Montreal Cognitive Assessment battery; MSA, multiple system atrophy; OH, orthostatic hypotension; N, number; NC, normal cognition; p, p-value; TMT-A, part A of Trail Making Test; T_0_, baseline; T_2_, follow up 2*.

**Figure 1:**
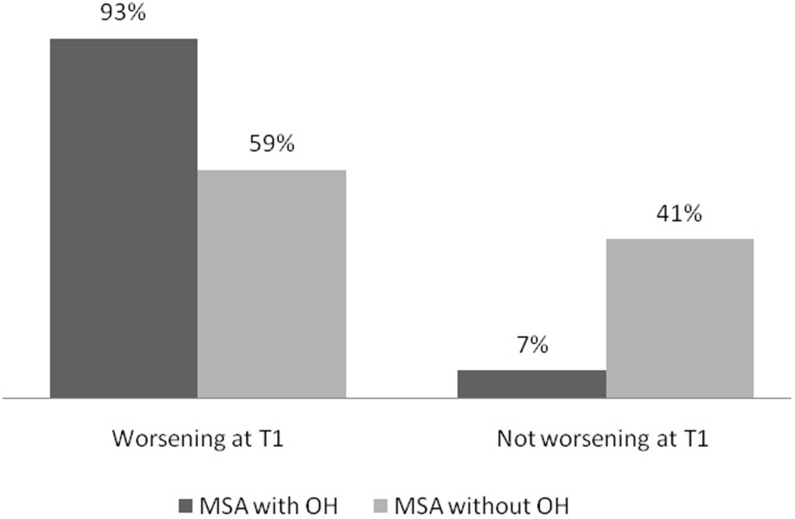
Percentage of MSA patients with and without OH according to change in global cognitive status at T_1_. MSA, multiple system atrophy; OH, orthostatic hypotension; T_1_, follow up 1.

The percentage of patients with worsened cognitive status at T_1_ was higher among patients with OH than among patients without OH (93 vs. 59%, *p* = 0.03) (Figure 1).

Logistic regression analysis showed that OH (β = −4.67, *p* < 0.01), education (β = 0.45, *p* = 0.02), age (β = 0.19, *p* = 0.03), and MOCA score at T_0_ (β = −0.26, *p* = 0.04) were significant predictors of global cognitive status worsening at T_1_, explaining 48% of the variance (*R*^2^ = 0.49), with OH accounting for 24% of variance. Gender (β = −0.04, *p* = 0.28), disease severity (β = −0.003, *p* = 0.32), and disease duration (β = −0.02, *p* = 0.27) did not affect cognitive worsening at T_1_.

As for different phenotypes, 16 patients with MSA-C and 16 with MSA-P did not differ by age (*p* = 0.24), education (*p* = 0.99), disease duration (*p* = 0.49), Unified Multiple System Atrophy Rating Scale (UMSARS)-II (*p* = 0.12), or UMSARS-IV (*p* = 0.19). MSA-C patients with OH (9/16 MSA-C patients) worsened more than MSA-C patients without OH (7/16) at T_1_ on semantic association test (*p* = 0.02) and AES (*p* = 0.04).

## Discussion

Cognitive disturbances in MSA range from mild single domain deficits to multiple domain impairment up to dementia in a minority of cases, but dementia is considered a non-supportive feature for the diagnosis of MSA (1). The severity of motor impairment may be a predictor of cognitive impairment in MSA (20–22), whereas the impact of OH on cognitive impairment has been poorly studied.

In our study, at baseline patients with OH differed from patients without OH only in a verbal repetition task, which indeed may be affected by speech disorders, motor programming, phonological encoding, and processing speed. At T_1_, we found that MSA patients with OH worsened on semantic memory and inhibition control skills more than patients without OH. More interestingly, by using cognitive categories, such as normal cognition (NC), MCI, and dementia, we found that almost all patients with OH worsened at 1-year follow-up on global cognitive status, suggesting that focusing on a single test is not very useful when assessing cognitive changes over time. Moreover, OH was an important predictor of cognitive worsening, together with education, age, and MOCA score, with OH accounting for half of the variance in the model. Our results are consistent with findings in patients with other α-synucleinopathies, such as Parkinson disease (PD) and dementia with Lewy body (DLB), where a relationship between OH and cognitive impairment has been described, as well as a significant negative effect of OH on postural instability and survival in these diseases (10). For example, PD patients with OH showed worse performances in Mini-Mental State Examination (MMSE) (23), verbal memory test (24), attention, visuospatial working memory and delayed verbal recall (25), and digit vigilance and visual episodic memory (26) compared to PD patients without OH. In a previous cross-sectional study (27) on 87 Korean drug-free patients with a diagnosis of early PD, cognitive impairment and dementia were significantly more prevalent among patients with OH than those without.

In a longitudinal study of 80 cognitively intact PD patients with a mean age of 66 years and mean disease duration of 5.7 years, the risk of developing dementia over a mean follow-up of 4.4 years was strongly associated with the drop in orthostatic systolic BP, and subjects having a systolic drop higher than 10 mm Hg showed a 7-fold increased dementia risk (28). Moreover, in a study including patients with DLB, but also Alzheimer's disease (AD) and AD with a vascular component, the MMSE score was lower in patients with OH than patients without OH (29).

In MSA, the time between any initial symptom and combined motor and autonomic dysfunction is a predictor of poor prognosis and shorter survival (30). Ueda et al. (31) found that the mean BP and the change in the mean BP at 60° tilt test were the only predictors of a MMSE score lower than 26 in MSA patients and suggested a careful observation of OH symptoms in order to detect cognitive dysfunction in patients with MSA. However, they did not analyze the differences in global cognitive performances between MSA with and without OH. In another study, MSA patients with OH scored lower on the only neuropsychological task assessed, i.e., modified Wisconsin Card Sorting Test, as compared to patients without OH, suggesting that OH was associated with frontal lobe dysfunction (32). On the other hand, Sambati et al. (33) found that 23 patients with MSA without cognitive impairment and 37 with cognitive impairment did not differ as for OH. Although they recognized that OH can modify the cognitive system, they did not divide the sample based on OH but used OH as a dependent variable. However, as for the mechanism underlying the possible association between OH and cognitive impairment, Udow et al. (34) proposed two hypotheses: first, the co-occurrence of OH and cognitive impairment may be a manifestation of diffuse neuropathological involvement reflecting parallel neurodegeneration of regions responsible for cardiovascular autonomic control and cognition (19); second, OH on its own or together with supine hypertension may have a negative impact on cognition in α-synucleinopathies, where the combination of cerebral hypoperfusion, with or without small vessel disease, may contribute to cognitive dysfunction (34).

In any case, the relationship between OH and cognitive impairment in PD is still controversial, since some authors have shown that, as reported for the elderly and patients with dementia, PD patients with OH were generally older than PD patients without OH, suggesting that age rather than OH may contribute to a decline in cognitive function (26, 35, 36). Similarly, O'Sullivan et al. (37) found no difference in time to develop cognitive impairment in MSA with early or late autonomic involvement.

On the other hand, our preliminary data may reflect more general findings in the middle-aged healthy population. In a review including 32 studies on OH and cognition in subjects with a mean or median age ≥65 years, 18 studies reported an association between OH and worse cognitive performance, and 14 reported no association. Interestingly, the studies using more than one cognitive task were more likely to find an association between OH and worse cognition, while the use of a single neuropsychological tool may underestimate this association (38). Furthermore, in a large longitudinal cohort of elderly subjects aged over 50 years, participants with OH showed a faster deterioration in executive and memory functions and presented a higher vascular burden as compared to participants without OH (39). However, it is still unclear whether the association between OH and poorer cognitive performance is causal, even in a general middle-aged population.

As for cognitive changes according to motor phenotypes, the finding of a worsening in semantic memory in MSA-C patients with OH can be ascribed to temporomesial cortex involvement (40), and, indeed, voxel-based morphometry MRI in MSA-C patients showed volume loss in the temporomesial cortex of both hemispheres as well as the left insular cortex as compared to healthy controls (41).

Regarding apathy, in literature there were few data about differences between MSA-P and MSA-C. Our preliminary data (42) showed that, independently from motor subtype, there was an increased prevalence of apathy over the course of MSA. A previous study (43) using the Neuropsychiatric Inventory (NPI) reported that there were no significant differences between MSA-P and MSA-C regarding neuropsychiatric symptoms, but that sleep disorders, apathy, and agitation/aggression were more severe in patients with MSA-C. However, Cao et al. (44) found that disease severity and MSA-C subtype are potential predictors of frontal lobe dysfunction. Therefore, patients with MSA-C might be more likely to have frontal lobe dysfunction than those with MSA-P, as reported also by Chang et al. (45). Additionally, the disruption of cerebellar-thalamo-cortical loops might also play a role in behavioral disorders (46, 47). This may account for the worsening of apathy in MSA-C as compared to MSA-P that was observed in our study.

We acknowledge that our study has some limitations, in particular, the short duration of the follow-up possibly affecting significant cognitive changes and the small sample at T_2_ evaluation. Moreover, our 2-year-follow-up data are preliminary due to the high percentage of patients lost at follow-up, which is justified by the fast progression of MSA.

In conclusion, further longitudinal studies on larger samples are necessary to confirm our preliminary data showing an association between OH and cognitive changes in MSA and to evaluate how chronic OH affects the long-term cognitive performance of patients with MSA.

## Data Availability Statement

The original contributions presented in the study are included in the article/supplementary material, further inquiries can be directed to the corresponding author/s.

## Ethics Statement

The studies involving human participants were reviewed and approved by Local Ethics Committee- Campania Sud. The patients/participants provided their written informed consent to participate in this study.

## Author Contributions

SC: substantial contributions to the conception or design of the work, analysis, interpretation of data for the work, and drafting the work. IC, AC, SS, MR, VA, and LF: the acquisition of data for the work. MA, MP, RE, and PB: revising it critically for important intellectual content. MP: substantial contributions to the conception or design of the work, analysis, and interpretation of data for the work, revising it critically for important intellectual content, and final approval of the version to be published. All authors contributed to the article and approved the submitted version.

## Conflict of Interest

Unrelated to this study, PB received consultancies as a member of the advisory board for Zambon, Lundbeck, UCB, Chiesi, Abbvie, and Acorda. RE received consultancies from Zambon and honoraria from TEVA. The other authors report no financial disclosures. The other authors report no financial disclosures. The remaining authors declare that the research was conducted in the absence of any commercial or financial relationships that could be construed as a potential conflict of interest.

## Publisher's Note

All claims expressed in this article are solely those of the authors and do not necessarily represent those of their affiliated organizations, or those of the publisher, the editors and the reviewers. Any product that may be evaluated in this article, or claim that may be made by its manufacturer, is not guaranteed or endorsed by the publisher.

## Abbreviations

5-RAWLT: Rey's auditory 15-word learning test
ADL: Basic Activities of Daily Life
AES: Apathy Evaluation Scale
BDI-II: Beck Depression Inventory
BJLO: Benton's Judgment of Line Orientation
BP: blood pressure
CDT: Clock Drawing test
DLB: dementia with Lewy body
DSM-5th: Statistical Diagnostic Manual of Psychiatry−5th Edition
F: female
IADL: Instrumental Activities of Daily Life
IQR: interquartile range
LEDD: total L-dopa equivalent daily dose
M: male
MCI: mild cognitive impairment
MDS: Movement Disorders Society
MMSE: Mini-Mental State Examination
MOCA: Montreal Cognitive Assessment battery
MSA: multiple system atrophy
MSA-C: multiple system atrophy with predominant cerebellar ataxia
MSA-D: multiple system atrophy with dementia
MSA-NC: multiple system atrophy with normal cognition
MSA-P: multiple system atrophy with predominant parkinsonism
N: number
NA: not applicable
OH: orthostatic hypotension
*p*: *p*-value
PD: Parkinson disease
SAND: Screening for Aphasia in NeuroDegeneration
SCWT: Stroop Color-Word Test
SPSS: Statistical Package for Social Science
TMT-A: part A of Trail Making Test
T_0_: baseline
T_1_: follow up1
T_2_: follow up2
UMSARS: Unified Multiple System Atrophy Rating Scale.
